# Extracellular Vesicles as Emerging Players in Intercellular Communication: Relevance in Mast Cell-Mediated Pathophysiology

**DOI:** 10.3390/ijms22179176

**Published:** 2021-08-25

**Authors:** Irit Shefler, Pazit Salamon, Yoseph A. Mekori

**Affiliations:** 1 The Herbert Mast Cell Disorders Center, Laboratory of Allergy and Clinical Immunology, Meir Medical Center, Kfar Saba 4428164, Israel; pazit.salamon@clalit.org.il (P.S.); ymekori@gmail.com (Y.A.M.); 2 Sackler School of Medicine, Tel Aviv University, Tel Aviv 6997801, Israel; 3 Tel Hai College, Tel Hai 1220800, Israel

**Keywords:** mast cell, extracellular vesicles, inflammation, cancer

## Abstract

Mast cells are major effector cells in eliciting allergic responses. They also play a significant role in establishing innate and adaptive immune responses, as well as in modulating tumor growth. Mast cells can be activated upon engagement of the high-affinity receptor FcεRI with specific IgE to multivalent antigens or in response to several FcεRI-independent mechanisms. Upon stimulation, mast cells secrete various preformed and newly synthesized mediators. Emerging evidence indicates their ability to be a rich source of secreted extracellular vesicles (EVs), including exosomes and microvesicles, which convey biological functions. Mast cell-derived EVs can interact with and affect other cells located nearby or at distant sites and modulate inflammation, allergic response, and tumor progression. Mast cells are also affected by EVs derived from other cells in the immune system or in the tumor microenvironment, which may activate mast cells to release different mediators. In this review, we summarize the latest data regarding the ability of mast cells to release or respond to EVs and their role in allergic responses, inflammation, and tumor progression. Understanding the release, composition, and uptake of EVs by cells located near to or at sites distant from mast cells in a variety of clinical conditions, such as allergic inflammation, mastocytosis, and lung cancer will contribute to developing novel therapeutic approaches.

## 1. Introduction

Mast cells (MCs) are derived from hematopoietic progenitor cells that enter almost all vascularized tissues, where they complete their maturation. [1,2,3]. MCs are known as essential effector cells in eliciting allergic responses and play a key role in innate and adaptive immunity. They are associated with a wide variety of pathophysiological processes, including tissue damage repair, thrombosis and homeostasis, autoimmune injury, and mastocytosis [2,4,5,6]. MCs are also often found at the site of tumors and are a component of the tumor microenvironment (TME). They function to promote or restrict tumor growth, depending on the mediators they release [5,7,8].

MCs are well-recognized by two kinds of highly expressed cell surface receptors: FcεRI, which is the IgE receptor, and c-kit (CD117), which is the stem cell factor (SCF) receptor. MCs can be activated by the binding of allergen-specific IgE via cross-linking of the FcεRI. MCs may also be activated via several FcεRI-independent mechanisms as well, including microbial products that signal through Toll-like receptors (TLR), direct injury, compounds known as basic secretagogues, IgG-antigen complexes, peptides, complement, proteases, and several cytokines and chemokines [9,10,11,12,13]. In addition to soluble mediators, we have shown that MCs can be activated by direct interaction with activated T cells or their microvesicles [14].

Upon activation, MCs can release a variety of bioactive mediators that are pre-stored in cytoplasmic granules (a process known as degranulation), including histamine, heparin, proteases, proteoglycans, and antimicrobial peptides. They can also selectively produce and release newly synthesized potent inflammatory mediators such as leukotriene C4 (LTC4), prostaglandin D2 (PGD2), and several pro- and anti-inflammatory cytokines and chemokines [9,10,15,16]. In addition to releasing different mediators, MCs secrete extracellular vehicles (EVs) spontaneously or in response to different stimuli [17].

EVs are membrane-surrounded structures that are secreted by various cell types. They are detectable in many biological fluids, including blood, urine, saliva, breast milk, amniotic fluid, ascites, cerebrospinal fluid, bile, and semen, as well as in conditioned media of cell cultures [18,19,20,21]. Once released to the extracellular space, EVs circulate in body fluids and modulate the metabolism of both neighboring and distant cells via the horizontal transfer of bioactive molecules, including proteins, lipids, DNA, RNA, and microRNA to recipient cells [22,23]. They have been implicated in several physiological and pathological processes, such as immune disorders, inflammation, neurological diseases, and cancer [19,24,25,26].

EVs are heterogeneous structures that differ from each other based on their biogenesis and size. They can be divided into three main subgroups: microvesicles, exosomes, and apoptotic bodies. Microvesicles range in size from 150 to 1000 nm and are formed by the outward budding and fusion of the plasma membrane.

Exosomes refer to smaller vesicles ranging from 30 to 150 nm in size. They are derived from the endosomal–lysosomal pathway by inward budding into endosomes, as intraluminal vesicles (ILV) within large multivesicular bodies (MVB), and then released to the extracellular space by fusion of MVB with the plasma membrane. Apoptotic bodies are heterogeneous vesicles. They vary from 50 to 5,000 nm in diameter and are released from cells undergoing apoptotic cell clearance [19,24,27].

Both EV subsets (microvesicles and exosomes) overlap in size and express some common identifying markers; making it more difficult to classify them. Therefore, guidelines published by the International Society for Extracellular Vesicles currently recommend using the term “EVs” when there is uncertainty about the subcellular origin of the vesicles [28]. In the present review, we chose to use the term “EVs” instead of the exact vesicle subset that was documented in the original articles.

Several lines of evidence suggest that EVs released by MCs may convey biological function by delivering them to distant target cells, thereby affecting inflammation, allergic response, and tumor progression. Furthermore, MCs can also be affected by EVs derived from other cells in the immune system or in the TME, which may lead to activating MCs to release different mediators [29]. In the present review, we discuss findings published in the last decade, using EVs and MCs as keywords. We focused on EVs derived from MCs and their potential roles in inflammation, allergic response, and cancer procreation, as well as the ability of EVs secreted from other cells to influence MCs in these conditions.

## 2. Mast Cells as the Origin of Extracellular Vesicles

### 2.1. Relevance of Mast Cell-Derived Extracellular Vesicles in Allergic Response and Inflammation

Data are continually emerging on the ability of MCs to exert interactions with other cells located in their proximity by secreting numerous mediators from their pre-stored granules and by releasing EVs [29,30]. MCs are able to release EVs either constitutively or upon stimulation. The composition of MC-derived EVs may differ in size, protein, lipid, and RNA and DNA content based on MC activation status and the type of MC stimuli. Thus, they have distinct functions [30,31,32].

Several reports have shown that MCs secrete EVs during FcεRI-induced degranulation [31]. Kormelink et al. showed that both mucosal-type MCs and connective tissue-type MCs, which represent the two main functionally different murine MC phenotypes, constitutively release EVs under resting conditions and have remarkably high amounts of EVs upon FcεRI cross-linking [17]. EVs derived from activated MCs were enriched with the EV marker CD63 and differed in their buoyant density, size, and lipid composition, as compared to EVs derived from resting MCs. Additionally, these activated MC-EVs contained high amounts of functional MC-specific mediators that were previously described as soluble only, as compared to EVs derived from resting MCs. Furthermore, activated MC-EVs release was found to correlate with β-hexosaminidase release [17]. Comparisons of the expression patterns of proteins, long non-coding RNAs, and miRNAs in EVs isolated from resting or FcεRI-activated bone marrow-derived mast cells (BMMCs) revealed that stimulated-EVs contained higher levels of tryptase, MC carboxypeptidase A, and IL-4 in contrast to resting-EVs [33]. The basic genomic features of the identified long non-coding RNAs and the length of the miRNAs differed between these two EV types, suggesting a potential regulatory function of MC-derived EVs [33]. However, the role of MC-derived EVs in the context of allergic reactions is still controversial. For example, EVs isolated from FcεRI-activated MCs displayed IgE and antigens on their surface, which enabled their efficient internalization by IgE-loaded MCs, mainly by endocytosis through the binding of antigens on EVs and IgE bound to MCs [34]. This internalization resulted in β-hexosaminidase release, a marker of MC degranulation, and induction of cytokine production at a level similar to that obtained by FcεRI cross-linking. These findings indicate a self-amplification mechanism that can contribute to exacerbation of local allergic reactions. FcεRI-mediated EVs uptake may also deliver antigens to other immune cells, such as FcεRI-positive dendritic cells (DC), further contributing to allergic responses [34]. Allergic diseases are characterized by increased serum levels of total IgE and specific IgE against common allergens, thereby activating the FcεRI on MCs. The sera of atopic individuals displayed varying amounts of EV-associated IgE that positively correlated with serum IgE levels, whereas IgE levels could not be detected on EVs purified from the sera of nonatopic donors. Moreover, only EVs derived from atopic individuals carried the FcεRI-α chain, supporting the assumption that these EVs display FcεRI/IgE complexes [34].

On the other hand, it has recently been shown that MC-derived EVs may have a protective role in allergic asthma. EVs derived from unstimulated BMMCs harbored FcεRI and could bind and neutralize free IgE in the serum, thus inhibiting MC activation by reducing its levels. In addition, in a mouse model of allergic asthma induced by exposure to ovalbumin, intravenous injection of MC-derived EVs eased the airway hyperresponsiveness over time. This improvement was accompanied by a remarkable decrease in ovalbumin-specific IgE in serum and histamine levels in bronchoalveolar lavage fluid. Changes in bronchoalveolar lavage fluid cytokine levels were also noticed. Furthermore, MC-derived EVs significantly modulated airway remodeling in allergic asthma, a suppressive effect that was positively correlated with treatment duration [35].

Communication between MCs and group 2 innate lymphoid cells (ILC2) in allergic inflammation occurs in the lungs, small intestine, and skin lesions of patients with atopic dermatitis. It has recently been demonstrated that interactions between MCs and ILC2 occurs via EVs. IgE-activated synovium-derived cultured MCs released EVs that can interact and be internalized into ILC2. The interaction of these EVs with ILC2 cells that were pre-activated with IL-33 led to enhanced production of type 2 cytokines such as IL-5 but not IL-13. Ekström et al. [36] and Valadi et al. [37] reported that MC-derived EVs contain miRNAs, which can be transferred to other cells and continue to function in these recipient cells. Indeed, the effect of MC-EVs on ILC2 could be attributed to miR103a-3p, which is highly expressed in EVs derived from activated MCs. The transfer of miR103a-3p to ILC2 via EVs resulted in the downregulation of protein arginine methyltransferase 5 expression, which led to enhanced IL-5 production. Furthermore, EVs from the sera of patients with atopic dermatitis express significantly higher levels of miR103a-3p than do EVs from the sera of nonatopic donors. Thus, miRs in EVs derived from human MCs following FcεRI aggregation might enhance the inflammation seen in atopic dermatitis [38].

Neuroinflammation is a response of the central nervous system to external stimuli, including surgery, infection, and toxins. It is manifested in part by the microglial activation and release of proinflammatory cytokines. Several studies have reported the central and peripheral MC critical roles in neuroinflammation by interacting with microglial cells to increase their migration and release of proinflammatory cytokines. An example of this interaction was found to occur via EVs released from LPS-activated MCs, which could transfer miR-409-3p to microglia, causing the downregulation of nuclear receptor subfamily 4 group A member (2Nr4a2) expression and activation of the NF-κB pathway, thereby promoting microglial migration, activation, and neuroinflammation [39].

In addition to miRs, EVs also contain and transfer extracellular RNA, such as ribosomal RNA, to recipient cells. Recently, it was established that EVs containing ribosomal RNA are released from MCs during exocytosis and degranulation. Thereafter, these EVs induced the release of pro-inflammatory cytokines such as MCP-1 and IL-6, as well as procoagulant responses in human umbilical cord vein endothelial cells in an extracellular RNA-dependent manner [40].

MCs can indirectly initiate the antigen-dependent T cell response by releasing EVs containing MHC class II and co-stimulatory molecules. Indeed, these MC-derived EVs had the capacity to induce B and T lymphocyte proliferation and cytokine production, which correlated with the induction of a Th1-type immune response, involving the production of IL-2, IL-12, and IFN-γ, but not IL-4 [29,41]. EVs derived from BMMCs enhanced the differentiation of naive CD4^+^ T cells to Th2 cells. This effect was found to occur, at least partially, by direct surface contact via ligation of OX40L present on MC-EVs with OX40 present on the surface of T cells, rather than by endocytosis [42].

Morphologic studies have documented an increase in the local density and activation of MCs in T cell-mediated inflammatory processes, as observed in psoriasis [43]. Indeed, a recent study has provided evidence that EVs derived from MCs are the source of cytosolic phospholipase A2 contributing to a CD1a-reactive T cell response in psoriasis patients [44].

Altogether, these findings provide evidence of the ability of MC-derived EVs to deliver certain messages and to act as mediators that contribute to immunomodulatory and/or immunoregulatory properties by interacting with other cells in distant sites.

### 2.2. Relevance of Mast Cell-Derived Extracellular Vesicles in Tumor Progression

Tumor progression is determined not only by the tumor cells themselves but also by their interactions with their surroundings, known as the TME. The TME has a dynamic composition that includes various cell types, such as immune cells, blood and lymphatic vessels, cancer-associated fibroblasts, tumor-associated macrophages, and myeloid-derived suppressor cells [45,46]. In the TME, the tumor cells communicate with each other and with stromal and immune cells via a sophisticated intercellular communication system through direct cell-to-cell contact or by classical paracrine signaling loops of cytokines or growth factors. MCs are often found in the microenvironment of several human solid and hematologic tumors and function as a component of the TME [7]. Accumulating data suggest that MCs have diverse roles in tumor biology. They were found to play both pro- and anti-tumorigenic roles in the TME depending on the tumor type and its developmental stage [5,7]. They can be pro-tumorigenic by affecting various events of tumor progression such as angiogenesis, proliferation invasiveness, survival, and metastasis. This can be mediated by releasing mediators such as histamine, prostaglandins, tryptase, β-FGF, TGF-β, VEGF, and IL-8. In contrast, the anti-tumorigenic effects of MCs include direct growth inhibition, immunologic stimulation, and decreased cell mobility. These effects involve the release of chymase, tryptase, TNF-α, and IL-9 [5,7,47,48,49].

Emerging data demonstrate that EVs are important in the mechanism of the cellular interchange of bioactive molecules. A number of studies have suggested that tumor cells communicate with each other and with neighboring microenvironmental cells via EVs, which coordinate various steps of tumor progression such as proliferation, angiogenesis, metastasis, and drug resistance [50]. Increasing evidence suggests that EVs derived from MCs have distinct functions in tumor progression. For example, EVs derived from HMC-1, a human neoplastic MC cell line, contain the KIT receptor, but not *c-KIT* mRNA. These EVs can be internalized into the A549 lung cancer cell line, which leads to enhanced proliferation and migration of the lung cancer cells by activating the PI3K/AKT signaling pathway [51]. Furthermore, EVs derived from MCs were also found to induce the epithelial to mesenchymal transition in these cells due to the induction of protein phosphorylation cascades known to be involved in this transition phenotype [52].

Several studies have explored the role of EVs derived from neoplastic MCs on systemic mastocytosis (SM). SM is a clonal disorder that harbors abnormal MC proliferation and pathological accumulation in several tissues, including bone marrow, lymph nodes, skin, liver, and spleen. SM displays various degrees of severity, from indolent to more aggressive forms. Among patients with SM, somatic mutations in *c-kit* that encodes for the KIT receptor have been detected in the bone marrow, as well as in skin and peripheral blood cells. The most common somatic mutation, Asp816Val (D816V), is located in the catalytic domain of KIT and results in augmented MC proliferation and survival [53]. The sera of SM patients contain high concentrations of small EVs that express hallmarks of MCs, such as FcεRI, MRGPRX2, tryptase, and activated KIT receptors. In addition, the concentration of these EVs correlated with other SM disease parameters such as serum tryptase. In liver biopsies obtained from SM patients, MCs infiltrate around hepatic portal areas, which is associated with development of liver fibrosis and other hepatic abnormalities [54]. Indeed, EVs derived from the sera of SM patients or from the human HMC-1 cell line affected the activation of hepatic stellate cells (HSC) by transferring active KIT from SM-EVs into the HSC cell line, resulting in proliferation, cytokine production, and differentiation of HSC, a process associated with liver pathology. The authors also demonstrated that injection of SM-EVs, but not EVs from healthy control subjects, into recipient mice resulted in increased α-SMA expression, a marker of HSC activation, around portal areas. These findings suggest a role for KIT within SM-EVs in the activation of HSC in vivo [55].

SM patients have high rates of osteoporosis and other bone diseases in association with the presence of MCs infiltrating bone marrow [56]. EVs derived from SM patients or from the HMC-1 MC cell line delivered miR-23a and miR-30a into pre-osteoblast cells. The internalization of these miRs via SM-EVs prevented osteogenic transcriptional programs, thus controlling their differentiation into osteoblasts, inhibiting bone production, and contributing to bone diseases, both in vitro and in vivo [57].

## 3. Mast Cells as Targets of Extracellular Vesicles

### 3.1. The Contribution of Extracellular Vesicles to Mast Cell-Induced Non-Allergic Responses

In addition to the function of EVs released by activated MCs, accumulated data published by our group and others have documented that EVs originating from various cell types can activate MCs [29].

The close physical proximity observed between MCs and T lymphocytes in inflamed tissues has raised the possibility of a functional interaction between these two cell populations. Indeed, morphologic studies have documented an increase in the local density of MCs and their activation during T cell-mediated inflammatory processes, as observed in cutaneous delayed-type hypersensitivity, sarcoidosis, inflammatory bowel disease, and rheumatoid arthritis [1,58]. We have documented that direct contact between MCs and activated T cells or their membranes is associated with Ras activation and sustained ERK phosphorylation, which results in MC activation and mediator release [59,60]. MCs were also found to be activated by EVs derived from activated T cells [14]. These T cell-derived EVs are actively internalized into MCs, a process that can be detected as early as one hour after initiation and appears to be completed after 24 h, resulting in both degranulation and release of specific cytokines such as Oncostatin M, IL-8, and IL-24 [14,61,62].

We also observed that EVs derived from these activated T cells contained high levels of miR-4443, which was delivered to MCs by the EVs. Mir-4443 serves as a negative regulator of the protein tyrosine phosphatase receptor type J (PTPRJ) gene, known to modulate the Ras signaling pathway by dephosphorylation of ERK [63]. Indeed, delivering miR-4443 into MCs by EVs-derived from activated T cells was found to augment ERK phosphorylation and IL-8 release. Thus, by delivering miR-4443, T cell-derived EVs may play an important role in MC activation within T cell-mediated inflammatory processes where MCs are involved [64].

Additionally, perivascular CD301b+ primed DC can shed antigen-bearing EVs after exposure to antigen. These antigen-loaded EVs were capable of trafficking to neighboring MCs and activating them to cause systemic anaphylaxis [65].

A recent study demonstrated that EVs derived from packed red blood cells (EVs-RBC) can activate human MCs, resulting in the increased expression of tryptase and PGD2 and the production of multiple inflammatory mediators, including IL-6, TNF-α, IL-4, INF-γ, CXCL-1, CXCL-5, and LTB-4. The activation of MCs with EVs-RBC also increased the levels of Toll-like receptor-3 (TLR-3) and MAPK activation. Inhibition of the MAPK pathway and TLR-3 resulted in the decreased production of inflammatory mediators, indicating that EVs-RBC activate MCs and elicit the production of multiple inflammatory mediators, partly via the TLR-3 and MAPK pathways. This observation may indicate that EVs-RBC can contribute to potentially harmful outcomes in recipients after blood transfusion by inducing MC activation [66].

Activated MCs participate in the chronic inflammation of cerebral arteries associated with intracranial aneurysm formation and rupture. Activated MCs can infiltrate into the aneurysmal walls and participate in the inflammatory response by releasing a wide range of inflammatory mediators that promote vascular destruction. In various models of organ injury, mesenchymal stem cells (MSC) were found to release EVs, which selectively accumulate at the lesion sites, where they mediate the processes of tissue repair and anti-inflammation by transferring proteins, lipids, and RNA into target cells [67]. Recently, it was demonstrated that MSC-derived EVs can internalize into MCs and prevent the rupture of intracranial aneurysms, in part through suppressing MC activation. The suppressive effect on MCs was mediated via a prostaglandin E2 (PGE2)-dependent mechanism. MSC-EVs increased the synthesis and release of PGE2 and upregulated the expression of the EP4 receptor on MCs, which may be associated with the anti-inflammatory response of MCs [68].

### 3.2. Mast Cells as Target of Extracellular Vesicles within the Tumor Microenvironment

In addition to tumor cells, a variety of other cells, such as stroma cells, fibroblasts, and immune cells, as well as the extracellular matrix and the network of blood-supplying vessels, together form the TME. Several studies have suggested that tumor-derived EVs influence a multitude of processes that aid in tumor progression, including angiogenesis, cellular proliferation, migration, invasion, metastasis, immunoediting, and drug resistance. This can be mediated by transferring bioactive cargos to recipient cells that are found in the TME. These bioactive materials can be comprised of markers and signaling molecules, oncogenic proteins, and nucleic acids, including various RNAs, such as microRNAs [50,69,70,71,72]. For example, tumor-derived EVs released from the non-small cell lung cancer (NSCLC) cell line A549 were shown to affect endothelial cells and stroma fibroblasts [73]. MCs are often found in the periphery of tumors and have been associated with both pro- and anti-tumorigenic features. The pro- and anti-tumorigenic role of MCs varied according to the tumor type and composition of the TME. Multiple features of the TME may affect the MC phenotype such as the SCF, hypoxia, accumulation of lactic acid, adenosine, PGE2, and low pH [74,75,76,77]. EVs derived from tumor cells are able to activate MCs to release several mediators [78,79,80]. Thus, EVs derived from NSCLC cells were internalized into MCs, in part by phagocytosis. The interaction of these EVs with MCs resulted in increased ERK phosphorylation, a rapid process that reached a maximal response at 1 min of activation and declined after 5 min. NSCLC-derived EVs stimulated MCs to release several mediators, such as TNF-α and monocyte chemoattractant protein 1 (MCP-1)/chemokine (C-C motif) ligand 2 (CCL2), as well as enhancing both their chemotactic and chemokinetic activity [78]. The activation of MCs by NSCLC-derived EVs may be due to the transfer of SCF found on EVs to the MCs, which leads to MC activation through SCF-KIT signal transduction [80].

In addition, EVs derived from pancreatic and NSCLC lung cancer cells can stimulate MCs to release several other mediators, including IL-8, IL-6, VEGF, and amphiregulin. This activation involves the autocrine formation of adenosine by a CD73-dependent mechanism and activation of the adenosine A3 receptor, leading to upregulation of tissue remodeling genes [79]. Thus, tumor-derived EVs provide a new possibility, in addition to soluble mediators in the TME, to activate MCs, which, in turn, can affect the cancerous process.

## 4. Concluding Remarks

**Figure 1:**
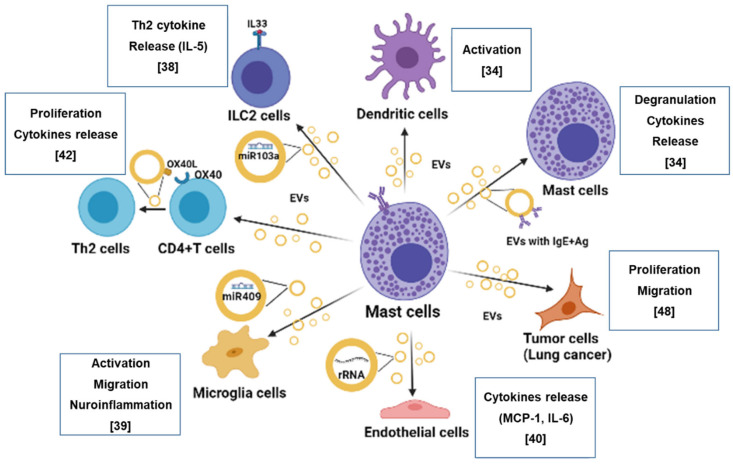
How MC-derived EVs communicate with other cells. The figure illustrates how MCs interact and communicate with other cells via EVs (as indicated in the references). Clockwise: EVs derived from MCs deliver antigens that are presented on their surface, to DC, contributing to allergic responses; EVs derived from FcεRI-activated MCs display IgE and antigens on their surface and induce degranulation and cytokine release from MCs; MC-derived EVs enhanced proliferation and migration of lung cancer cells; ribosomal RNA is delivered to endothelial cells by MC-derived EVs and induces the release of pro-inflammatory cytokines; miR-409-3p is carried and transferred by MC-EVs to microglial cells, increasing their migration and release of pro-inflammatory cytokines; MC-derived EVs carry OX40L, which interacts with OX40 present on the surface of T cells, enhancing the differentiation of naïve CD4+ T cells into Th2 cells; EVs derived from MCs deliver miR-103a to IL-33-activted ILC2 and induce IL-5 release. The figure was created with BioRender.com.

In addition to being a source of various mediators in response to different stimuli, MCs have recently captured considerable attention as an important source of EVs implicated in many physiological and pathological processes. EVs generated by stimulation differ in terms of size, morphology, and molecular profiles depending on the MC stimulus, in addition to differing from EVs derived from unstimulated MCs. EVs released from MCs can interact with other cell types located in their proximity or at distal sites, including immune cells (T, B, and DC), neurons, and vascular cells (see Figure 1). They act as a vehicle for a wide variety of biological cargo, including lipids, DNA, RNA, and microRNA, to recipient cells. These interactions may influence allergic reactions and inflammation. Furthermore, increasing evidence suggest that MC-derived EVs also have a distinct function in tumor progression. EVs derived from MCs located in the TME can increase the proliferation and migration of tumor cells, as well as induce angiogenesis, stimulate antitumor immune response, and recruit fibroblasts and macrophages [81].

**Figure 2:**
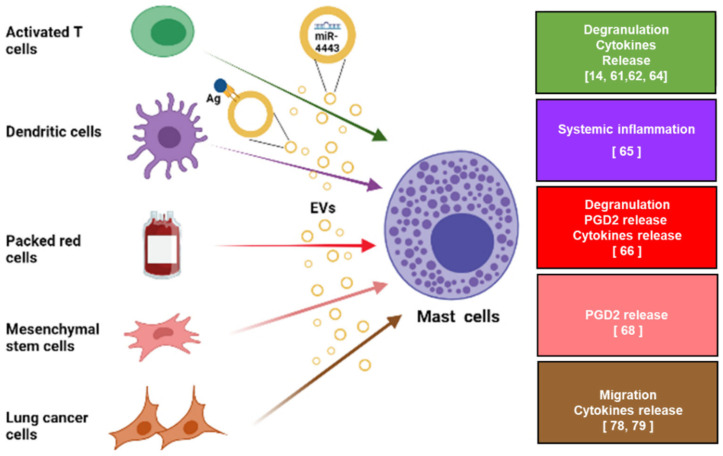
The effect of EVs originating from various cell types on MC activation. MCs may be a target for EVs originating from various cell types. From top to bottom (The colors of the arrows refer to the colors of the boxes): activated T cells release EVs that induce MC degranulation and cytokine release in part by carrying and transferring miR-4443 to MCs; perivascular CD301b+-primed DC can shed antigen-loaded EVs that bind to MCs and activate them; EVs derived from packed red blood cells can activate MCs, resulting in degranulation and release of PGD2 and cytokines; MSC-derived EVs induce the release of PGE2 from MCs; EVs derived from lung cancer cells induce MC migration and cytokine release. The figure was created with BioRender.com.

Accumulated data also reveal that MCs may serve as a target for EVs that originate from various cell types (see Figure 2). In the context of inflammatory diseases, MCs can be activated by EVs derived from activated T cells and DC, thus leading to MC degranulation and cytokine release, in part by delivering miR (such miR-4443) via T cell derived-EVs.

The presence of MCs in the TME has been well-documented [47]. However, one important question is whether EVs derived from tumor cells can activate MCs. Indeed, our group and others have shown that EVs derived from tumor cells can activate MCs, resulting in enhancing their migration ability and release of specific cytokines.

The possibility that MCs interact with other cell types via EVs may contribute to understanding how MCs communicate with nearby and with distant cells and their contribution to various diseases, as described above. This may help in the development of novel therapeutic modalities. Furthermore, due to the bioactive cargoes of EVs (e.g., miRNAs, nucleic acids, or proteins) and their distribution in most body fluids [82,83,84], they may provide a promising source of diagnostic biomarkers for MC-related diseases. Indeed, EVs were found to serve as biomarkers in cancer [85], asthma [86], Alzheimer’s [87] and kidney disease [88]. However, several limitations, including the lack of sensitive purification method, storage stability, low yield, and purity, limit EV’s clinical applications. New EV screening techniques and storage preservation technologies, which are under development, will help to overcome the challenges associated with EV isolation and processing [82,89].

Finally, MCs have been implicated in the pathogenesis of the cytokine storm in COVID-19 [90,91]. It will be interesting to verify whether EVs cause this MC activation.

## Abbreviations

BMMC	Bone marrow-derived mast cell
CNS	Central nervous system
DC	Dendritic cell
EMT	Epithelial-to-mesenchymal transition
EVs	Extracellular vesicles
ERK	Extracellular signal-regulated kinase
HSC	Hepatic stellate cell
ILC2	Group 2 innate lymphoid cell
LTC4	Leukotriene C4
MC	Mast cell
MSC	Mesenchymal stem cell
NSCLC	Non-small cell lung cancer
PGD2	Prostaglandin D2
PGE2	Prostaglandin E2
SCF	Stem cell factor
SM	Systemic mastocytosis
TLRs	Toll-like receptors
TME	Tumor microenvironment
